# Tunable Whispering Gallery Mode Photonic Device Based on Microstructured Optical Fiber with Internal Electrodes

**DOI:** 10.1038/s41598-019-48598-z

**Published:** 2019-08-19

**Authors:** Tatiana Muñoz-Hernández, Erick Reyes-Vera, Pedro Torres

**Affiliations:** 1 Escuela de Física, Universidad Nacional de Colombia, sede Medellín, Medellín, Colombia; 2 Department of Electronic and Telecommunications Engineering, Instituto Tecnológico Metropolitano, Medellín, Colombia; 3 Department of Electrical and Electronic Engineering, Universidad Nacional de Colombia, sede Bogota, Bogotá, Colombia

**Keywords:** Microresonators, Photonic devices, Fibre optics and optical communications

## Abstract

We propose and experimentally demonstrate the first tunable whispering gallery mode (WGM) photonic device based on side-hole microstructured optical fiber (SH-MOF) with internal electrodes, in which the WGM quality factors do not decrease significantly during the tuning process. The resonant modes are redshifted simply by increasing the temperature. A description of the thermal tuning properties of the WGMs in SH-MOF with internal electrodes is performed by using a two-stage computational methodology, where the effects of metal filling process are considered. SH-MOF devices with internal electrodes are tested and the experimental results show excellent agreement with the theory. A linear relationship between the shift rate of the WGM modes and temperature is observed. The tunable SH-MOF microresonator with internal electrodes is anticipated to find potential applications in optical filtering, optical switching, and highly integrated tunable photonic devices.

## Introduction

Over the past few decades, whispering-gallery microresonators (WGRs) have attracted considerable interest and, after the development of efficient and robust coupling using evanescent field couplers, such as prisms and tapered optical fibers, the realization of the WGR as a useful optical device became apparent^1,2^. Today, WGRs can be found in a number of different applications, including cavity-enhanced nonlinear optical^1,3^, low-threshold narrow-linewidth microlasers^4,5^, spectroscopy^6,7^, optical communications–switching, filtering, and multiplexing^8–10^–and sensing^1,2,6,11–13^. Conventional circular structures such as microspheres, microdisks, microtoroids, and microcylinders, manufactured by molding a wide variety of materials from polymers and crystalline materials to different types of glass, are often selected to serve as high quality-factor optical microcavities, in which light resonates as whispering-gallery modes (WGMs) by total internal reflection at the interface between microcavities and their surrounding media. A change in the surrounding medium or a change in the properties of the cavity may cause a shift in the resonant spectrum which can be calibrated for sensing purposes^7,11–13^. For example, WGRs have been studied extensively over the last two decades in the field of chemical and biochemical sensing, showing remarkable detection capabilities down to the single virus, single nanoparticle, and single molecule level^11–13^.

The ability to tune the WGM microresonators is a key requirement for many applications. Several techniques for tuning solid-state microcavities have already been explored, e.g., size reduction by etching process^14,15^ or gradual changes in the geometry of the microcavitiy through thin film deposition^15^ allow precise and simple tuning of the WGM resonances, however, cause irrecoverable changes in the microcavity and provide irreversible tuning. Most tunable microcavities reported in the literature are based on a variation of the refractive index of the cavity material, either by electro-optical, thermo-optical or mechanical-strain/pressure modulation. Electro-optical tuning approach can achieve around 40 MHz (tenths of a picometer) per volt in lithium niobate-based platforms^16^ and around 800 MHz (some picometers) per volt in electro-optic polymer-based platforms^17^. Thermo-optical variation of WGM cavities is typically applied in platforms based on polymers with relatively large thermo-optic coefficient with a possible detuning of the resonance wavelength of approximately 50 GHz (a few hundred picometer) per Kelvin^18^. Recently, photothermal tuning of microcavities have been reported. For example, a sphere microresonator fabricated from an Er:Yb co-doped phosphate glass^19^ is pumped at 978 nm and the heat generated by absorption of the pump expands the cavity, thereby altering the cavity size and refractive index. Linear (∼488 GHz) and strong nonlinear (∼700 GHz) tunings were realized, which are limited by the melting point of phosphate glass. Besides, based on the strong photothermal effect, a magnetic-fluid-filled microcapillary resonator is demonstrated^20^. A tuning sensitivity up to 0.15 nm/mW over a tuning range of 3.3 nm was achieved. However, magnetic fluids have a strong absorption of light and this decreases performance. Mechanical-strain/pressure tuning, which includes squeezing or stretching microsphere^7,21–23^, microbubble^24^ and bottle microresonators^25^, allows flexibility and wide tuning ranges (300 GHz–700 GHz); however, these techniques involve sophisticated equipment, therefore making them difficult to implement.

Tuning schemes can be more functional with the use of a suitable optical platform. Cylindrical microstructured optical fiber (MOF) microresonators infiltrated with fluids provide an alternative approach to tunability. Electrical^26^ and magnetic^27^ tuning of MOF-based microresonators integrated with nematic and ferromagnetic liquid crystals, respectively, has been demonstrated and electrical and magnetic tuning sensitivities up to −0.01 nm/V and −39.6 pm/mT are observed in tuning ranges of 0.527 nm and 2 nm, respectively. However, both approaches deteriorate the Q factors of microcavities and obviously these schemes are not suitable for applications in which Q factors are required.

In this work, we propose the use of MOF-based microresonators but in a drastically different configuration based on metal-filled side-hole MOF (SH-MOF) for thermo-optical tuning. When heat is transferred to the SH-MOF, the metal expands and squeezes the fiber, which alters the cavity size and the refractive index and, therefore, tunes the resonant frequency of the fiber microcavity. In this scheme, WGMs do not interact directly with the filler metal. Consequently, the Q factor of the silica microcavity do not decrease significantly during the tuning process. Various metals with a relatively low melting point can be used for this purpose. For example, recently we studied the birefringent optical properties of a side-hole MOF filled with Bismuth (Bi) and Indium (In)^28–30^. The modulation in the fiber birefringence is accomplished through the thermal-stress field induced by the expansion of the metal-filled holes. In this report, Bi with a small thermal expansion coefficient, α_*m*_ ≈ 13.2 × 10^−6^ K^−1^ ^28^ and In, with a large thermal expansion coefficient, α_*m*_ ≈ 32.1 × 10^−6^ K^−1^ ^28^, are used to investigate the effect of the filler metals on the tuning response of the active microcavity. Note that our platform has remarkable advantages such as ease of manipulation and high tuning sensitivity with a large tuning range, which ensure its potential applications in optical filtering, optical switching, and highly integrated tunable photonic devices.

## Materials and Methods

### Working principle

As already mentioned, WGMs occur when light is trapped inside a microresonator by total internal reflection, circulating along the inner surface and returning in phase after single or multiple round trips to satisfy the resonance condition. When a theoretical analysis is carried out by solving Maxwell equation with the corresponding boundary conditions, it is found that WGMs could be classified into two different polarization components, Transversal Magnetic (*TE*^*z*^) mode and Transversal Electric (*TE*^*z*^) mode, where the electric field oscillates parallel to or perpendicular to outer surface of the microresonator, respectively^31–35^. The longitudinal component of the magnetic field for a *TE*^*z*^ WGM is given by^36,37^:

1 $${H}_{z}={e}^{-jm\phi }{e}^{j\omega t}\{\begin{array}{c}{A}_{1}\,{J}_{m}({k}_{1}\rho )+{A}_{2}\,{Y}_{m}({k}_{1}\rho ),\rho  < b\\ {A}_{3}\,{H}_{m}^{(2)}({k}_{2}\rho ),\rho  > b\end{array}$$

with ${k}_{i}=\frac{2\pi }{\lambda }{n}_{i}$; *i* = 1, 2 and, where *J*_*m*_ and *Y*_*m*_ are the Bessel functions of first and second kind, respectively, ${H}_{m}^{(2)}$ are the Hankel functions, *m* is the angular order, *A*_1_, *A*_2_ and *A*_3_ are the amplitude coefficients, *n*_*i*_ is the refractive index of each medium, and *λ* is the wavelength. The longitudinal component of the electrifield for a ${TM}^{z}$ WGM can be expressed similarly.

An exact analytic solution of the corresponding electromagnetic boundary problem is generally not possible, so the resonance frequencies must be searched numerically or by means of analytical approximations. For example, to theoretically analyze the WGM transmission characteristics, we have calculated the resonant wavelength, expressed as ${\lambda }_{m}^{l}$, where *l* is the radial mode order, by using the asymptotic formula for cylindrical microcavity^26,34,38,39^.

2 $$n\frac{2\pi {r}_{0}{n}_{2}}{{\lambda }_{m}^{l}}=m+{2}^{-1/3}{a}_{l}{m}^{1/3}-\frac{P}{{({n}^{2}-1)}^{1/2}}+\frac{3}{10}{2}^{-2/3}{a}_{l}^{2}{m}^{-1/3}-\frac{{2}^{-1/3}P({n}^{2}-2{P}^{2}/3)}{{({n}^{2}-1)}^{3/2}}{a}_{l}{m}^{-2/3}+O({m}^{-1})$$

where *r*_0_ is the radio of the resonant cavity, *n*_1_ is the index for the cavity, *n*_2_ the index for the medium around the cavity, respectively; *n* = *n*_1_/*n*_2_, *P* = *n* for *TM* wave, while *P* = 1/*n* for *TE* wave; *a*_*l*_ is the root of Airy function.

To exploit WGMs, the modes are excited by evanescent field coupling between a phase matched tapered optical fiber and the microresonator, and scanning across a narrow wavelength range in the transmission spectrum at the output end of the fiber to identify the resonance positions and linewidths, typically with very high quality factors, $Q={\lambda }_{m}^{l}/\delta {\lambda }_{m}^{l}$ ($\delta {\lambda }_{m}^{l}$ is the linewidth of the detected mode). The capability to tune the resonant frequencies in optical microcavities is a key feature for many applications. A generalized expression to obtain the resonance spectral shift of the cavity as a function of the temperature is given by^19,38–41^:

3 $${\rm{\Delta }}{\lambda }_{RS}=\lambda (\frac{1}{n}\,\frac{dn}{dT}+\frac{1}{L}\,\frac{dL}{dT}){\rm{\Delta }}T,$$

where the first term is due to the induced refractive index changes as a function of the temperature. In our case, this term depends on both photo-elastic and thermo-optic effects. On the other hand, the second term depends of the variation of the perimeter of the cavity, which is given due to the mechanical variation of the size of our cavity when it is submitted to thermal variations.

### Metal filling process

**Figure 1:**
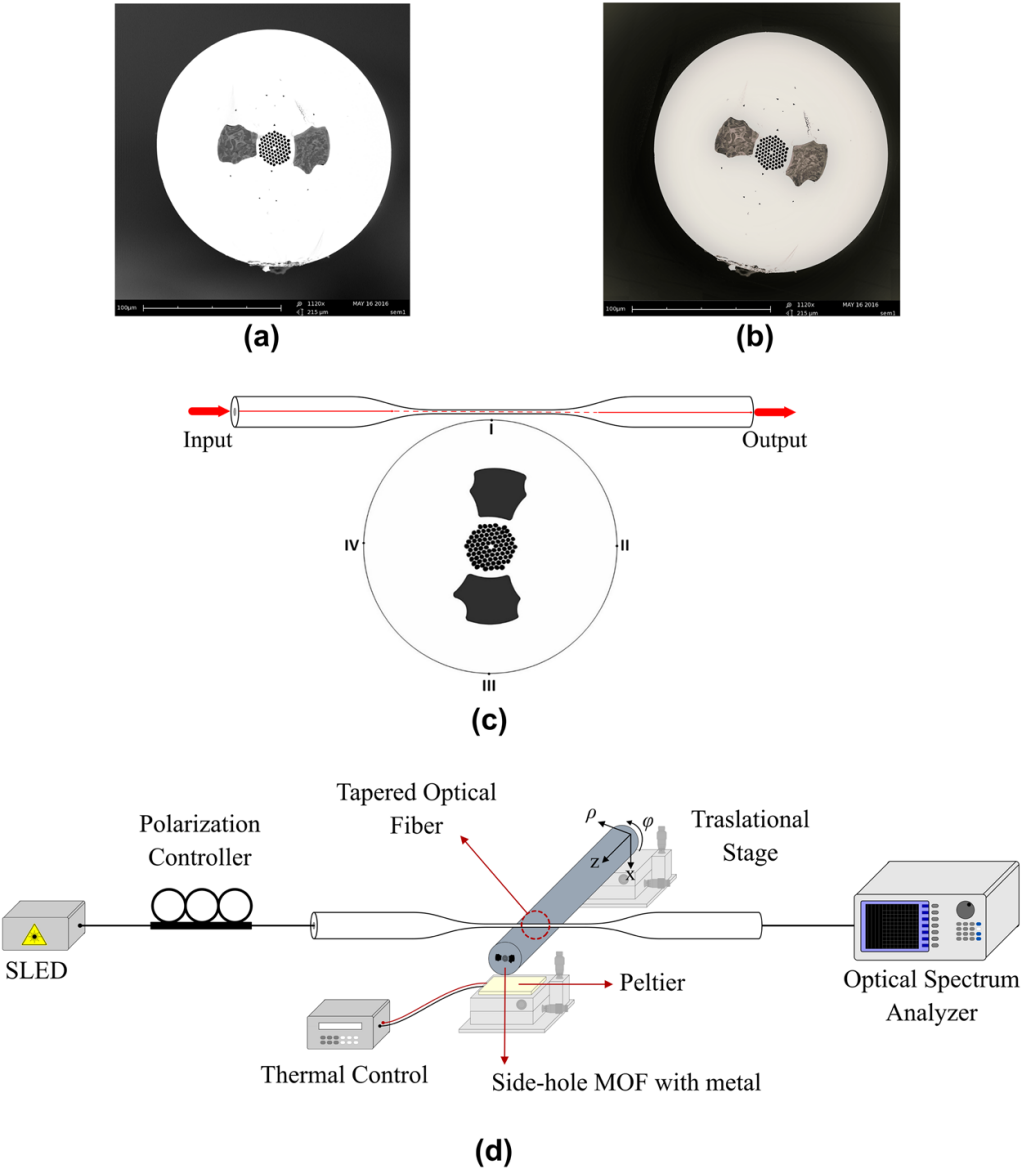
Tunable whispering gallery mode device based on side-hole MOF with internal electrodes. (**a**) SEM image of side-hole MOF with Bi electrodes. (**b**) SEM image of side-hole MOF with In electrodes. (**c**) Diagram of interaction between the fiber taper and the MOF-based microcavity¸ where the four points of interaction considered in the study are indicated. (**a**–**c**) adapted with permission from^42^, Optical Society of America. (**d**) Schematic diagram of the experimental setup developed to study the WGM photonic device. During each measurement of the WGM shift with respect to the temperature, it is verified through CCD images that the fiber taper is in direct contact with the MOF at the contact point to be studied and, on the other hand, the input polarization state was fixed in order to excite separately *TE*^*z*^- or *TM*^*z*^-polarized WGMs.

SEM images of the SH-MOFs used as a microcavity are illustrated in Fig. 1a,b. With an external diameter of approximately 170 μm, the fused silica MOF has two large holes having a width of approximately 23 μm and approximately 15 µm separates them from the edges of the core. The metal filling process is similar to what is reported previously^28,29^. Briefly, the filler metal is placed inside a pressure chamber, which is heated using an oven to a temperature slightly above its melting point (170 °C and 350 °C for In and Bi, respectively). One end of the SH-MOF is immersed into the molten metal, and the other one is placed out of the oven. A pressure of 3 bar is applied to inject the melted metal into the side holes of the MOF, and then self-cooled with the chamber turned off and close. The two metals used in this study were well incorporated by fully filling the side holes of the MOF to introduce the large thermal mismatch with silica glass (α_*m*_ ≈ 0.5 × 10^−6^ K^−1^). The advantages of the filling procedure are that it is easy to perform using metals with a low melting point and it provides continuous metal wires, which was completely demonstrated in previous works^28,29^. Note that the maximum operating temperature of this type of devices is limited by the melting point of filler metals. When the metal is completely melted, only minor temperature dependence remains, since the thermal-induced stress from the metal in the holes is relaxed^42^.

### Experimental setup

Figure 1d shows a scheme of the experimental arrangement, where a fiber adiabatic taper with a waist diameter of ∼1.5 μm and a length of ∼1 cm was employed to excite and interrogate the WGM resonances. The optical fiber tapering is undertaken using the flame-brushing technique from the single mode fiber (SMF-28, Corning Inc), by adjusting the control parameters of the drawing platform. A superluminescent light emitting diode (Exalos, EXS1520-2111) with a wavelength range of 1500–1600 nm was used as light source. A polarization controller was inserted after the light source to adjust the polarization state of the light in order to excite separately $T{E}^{z}$- or $T{M}^{z}$-polarized WGMs. The transmitted light spectra are recorded using an optical spectrum analyzer (OSA) (Yokogawa, AQ6370B) set at a wavelength resolution of 0.02 nm. During each measurement the metal-filled MOFs is in contact with the fiber taper waist positioned perpendicular to the axis of the fiber microcavity, whereby the system is strongly overcoupled^27,43^. In each test a thermal control was carried out, in which a temperature sensor (DS18B20) adapted to an acquisition program in ARDUINO UNO was implemented, performing temperature variation in a range from 25 °C to 60 °C, limited by technical shortcomings, by using a heating system based on a semiconductor Peltier cooler. Ohmic heating of the metal filled holes has also proved to be an effective means of controlling the optical properties of the fiber, accessing one of the internal electrodes by lateral polishing until the inserted metal is exposed, followed by its connection to an external circuit^44^. With our heating system, the fiber structure is sufficiently robust and uniform thermal conditions are ensured.

### Computational methodology

In order to investigate our tunable WGM photonic device based on metal-filled side-hole MOF, a model based on the finite element method (FEM) by using COMSOL Multiphysics was developed, which has been previously validated with experimental results^28,29,42,45^. The thermal expansion of the electrodes will cause both a change in the geometrical shape of the microresonator (due to mechanical strain) and a change in the refractive index of fused silica (due to mechanical stress), inducing a WGM shift.

Unlike other fiber configurations in which the structure expands homogeneously^46^, the distribution and geometry of both the metal electrodes and fiber microstructure in Fig. 1a,b show the complexity of the physical problem to be solved, which has an important effect on the interaction of the SH-MOF microresonator with the fiber taper as illustrated in Fig. 1c. Importantly, our computational methodology consists of two stages. In the first stage, the strain and stress components generated in the SH-MOF during the metal filling process are computed. In this process, there are four important parameters: the thermal expansion coefficients of silica and the filler metal, the melting point of the metal and the applied pressure in the metal filling process. In the second stage, the deformation in the SH-MOF cross section is accomplished through the thermal-stress field induced by the interplay between the residual stress induced by the metal filling process and the stress induced by the thermal expansion of the metal with the temperature. In both stage it is necessary to implement the photo-elastic theory to obtain the principal refractive indices (*n*_*x*_, *n*_*y*_, *n*_*z*_) in whole structure. The change of refractive index with the applied thermal stress is given by the Neumann–Maxwell equation as:

4 $$\begin{array}{rcl}{n}_{x} & = & {n}_{0x}+{B}_{1}{\sigma }_{x}+{B}_{2}({\sigma }_{y}+{\sigma }_{z}),\\ {n}_{y} & = & {n}_{0y}+{B}_{1}{\sigma }_{y}+{B}_{2}({\sigma }_{x}+{\sigma }_{z}),\\ {n}_{z} & = & {n}_{0z}+{B}_{1}{\sigma }_{z}+{B}_{2}({\sigma }_{x}+{\sigma }_{y}).\end{array}$$

here, *n*_0*x*_, *n*_0*y*_, *n*_0*z*_ are the principal refractive indices for the unstressed material and *σ*_*x*_, *σ*_*y*_, *σ*_*z*_ are principal stress components, which depends on the applied temperature. *B*_1_ and *B*_2_ are stress optic coefficients of silica. The details of the all material parameters can be found in^28^.

## Results

### Simulation results

**Figure 2:**
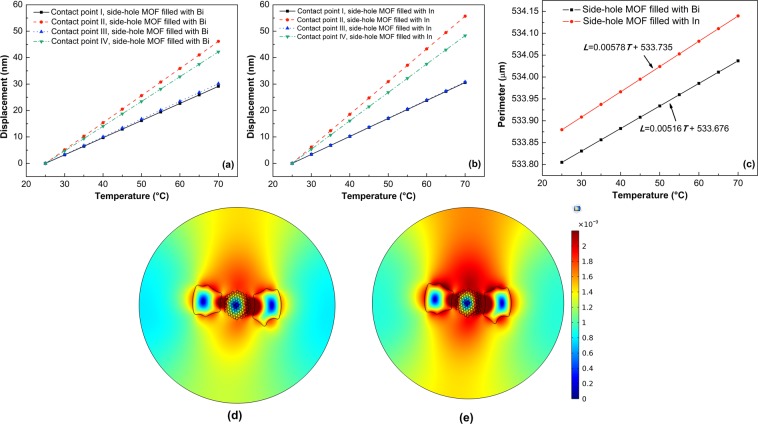
Thermoelastic analysis of side-hole MOF with internal electrodes subjected to temperature changes. Displacement of the four contact points indicated in Fig. 1c as a function of temperature: (**a**) side-hole MOF with Bi electrodes, (**b**) side-hole MOF with In electrodes. **(c)** Variation of fiber perimeter as a function of temperature for both metal-filled cavities. (**d**,**e**) Displacement maps, measured from its original position at room temperature, of the side-hole MOF with In electrodes at 35 °C and 70 °C, respectively.

The tunability of microresonator modes is governed not only by the resonator itself, but also, as in this case, by the interaction of the metal-filled SH-MOF with the fiber taper. That is why we first studied the displacement—measured from its original position at room temperature—of the four contact points specified in Fig. 1c when the temperature applied to the microresonator changes from 25 °C to 70 °C. These points are the most interesting to study since in I and III the taper is close to the electrodes, while in II and IV the taper is farther from the electrodes. Figure 2a,b show the obtained results for the SH-MOF filled with Bi and In, respectively. The results reveal, as expected, a very small change in the eccentricity of the fiber, which gives a relatively small contribution to total tuning, hence the conclusion about the contributions related in (3) holds. As depicted in Fig. 2a,b, both cavities show the smallest and largest displacements, respectively, at contact points I and II, as a result of the expansion of both electrodes, as can be corroborated by the displacement map in Fig. 2d,e. On the other side, Fig. 2c shows the thermal-induced deformation experienced by the microresonator, in which a linear dependence of the perimeter of the cavity on the temperature is revealed. It is worth noting that our computational methodology accounts for the effects of the filling process to the SH-MOF according to the filler metal, so it was expected that the initial conditions at room temperature in Fig. 2c are different. From this figure one can observe that for each 1 °C of increase in temperature it is found that the perimeter of the cavity experiences an increase of 5.156 nm for the cavity with Bi and 5.776 nm for the cavity with In. It is clear that the magnitude and distribution of stress/strain are governed by the distribution of the holes in the microstructure and depend strongly on the filler metal. Due to its large thermal expansion coefficient, the In-filled SH-MOF cavity undergoes greater deformation, which should expect that this type of microcavity has a lower quality factor *Q* because of the asymmetry induced by the thermal expansion of the electrodes. The deformation of the MOF from its circular geometry increases the leakage of the mode field out of the resonator. This leakage occurs at the points of higher curvature of the resonator contour (regions around points II and IV)^47,48^; see Fig. 1a,b.

**Figure 3:**
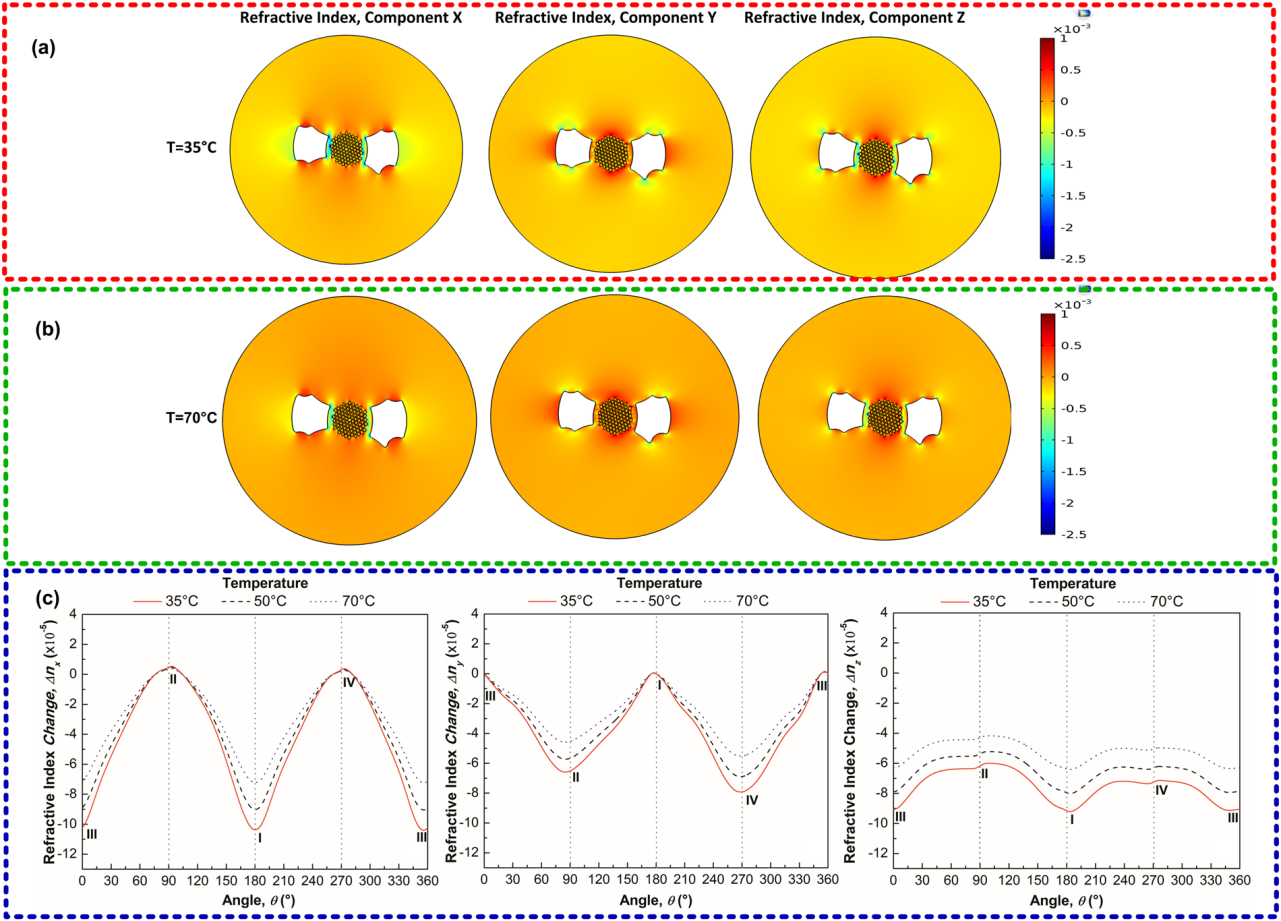
Refractive index changes within the side-hole MOF with internal electrodes subjected to temperature changes. (**a**,**b**) Refractive index changes of the side-hole MOF with In electrodes at 35 °C and 70 °C. (**c**) Refractive index changes along de perimeter of the SH-MOF against temperature. It is clear that for the transverse directions (*n*_*x*_ and *n*_y_) the refractive index change is greater in comparison to the longitudinal direction (*n*_z_), as a consequence of the different mechanical stress components within the material of the resonator due to the thermal expansion of the internal electrodes of the MOF. In (**c**) the angle is measured counterclockwise from point III (θ = 0°), whereupon the other three points are at θ = 90° (Point II), θ = 180° (Point I) and θ = 270° (Point IV).

As stated in Eq. (3), the second aspect to be considered in the WGM tuning is a possible change in the refractive index caused by different mechanical stress components within the material of the resonator. The use of Eq. (4) allows us to obtain the distribution of the refractive index change within the cavity as illustrated in Fig. 3. It can be seen in Fig. 3c that the refractive index changes in the perimeter of the metal-filled SH-MOF microcavity are space-dependent and decrease with the increase in temperature, as a consequence of the different stress components within the material of the microresonator due to the thermal expansion of the internal electrodes of the MOF^28,29^.

**Figure 4:**
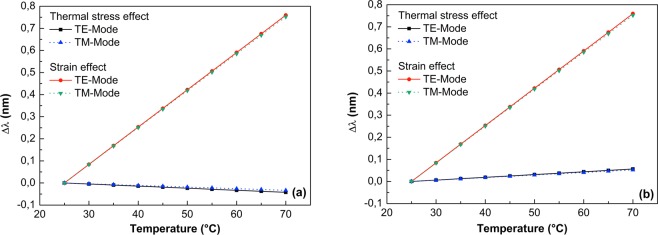
Comparison of the capability of thermal stress and strain effects to tune the WGM resonant of the microresonator based on SH-MOF with In electrodes. (**a**) WGM tuning analysis at the contact point I. (**b**) WGM tuning analysis at the contact point II.

In order to evaluate the contribution of thermal stress (change in the refractive index) and strain (expansion of the microcavity) effects on the WGM shift, Fig. 4 shows the contribution of both terms when the taper is placed at the contact points I and II. From these results, it is evident that the WGM shifts mainly depend of the strain effect, which is at least 15 times larger than the thermal stress component, and that the tunability of the TE and TM modes do not differ appreciably. As can be seen in this figure, the WGM shift analysis also reveals that at the contact point I the thermal stress effect is a negative component while at the contact point II it is positive; the strain effect is the same for all cases. The fact that high-intensity regions in the WGM field of the deformed MOF are located at the points of the highest curvature of the contour^49^, where the WGM field penetrates further into the surrounding media and, therefore, the coupling of the fiber taper to the MOF microcavity changes the effective refractive index and frequency of the WGM in the resonator and, therefore, shifts the WGM resonance. Hence, it is expected that the total WGM shift at contact point I is slightly less than the total WGM shift at point II^49^. As already mentioned, this remarkable difference, unlike the cavities that expand homogeneously^46^, shows that in this case the tunability of the WGMs is governed by the interaction of the metal-filled SH-MOF microresonator with the fiber taper. In the next section the theory results are applied to the problem of metal-filled SH-MOF microresonator and we check the validity of this computational methodology.

## Experimental Results

**Figure 5:**
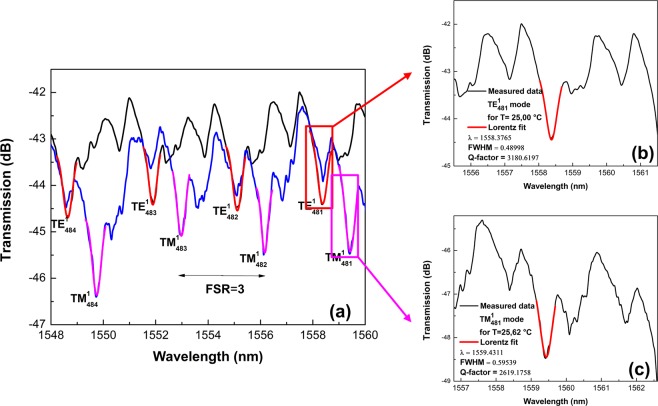
Experimentally acquired WGM transmission spectra and corresponding Lorentzian fitting to estimate the Q-factor for the microresonator based on SH-MOF with Bi electrodes. (**a**) *TE* and *TM* WGM resonances at room temperature with the fiber taper at contact point II. (**b**,**c**) Lorentzian lineshape of the *TE* and *TM* WGM resonances with estimated Q-factors.

By adjusting the polarization state of the incident light, we have experimentally acquired the WGM transmission spectra for *TE* and *TM* modes. As an example, Fig. 5a shows the experimental transmission spectra for the Bi-filled SH-MOF microresonator at room temperature with the fiber taper at contact point II. As can be seen from the Fig. 5a, the spectra contain a quasi-periodic structure of dips. The good agreement with Equation (2) identifies these resonances as the whispering gallery modes. The spectra show two families of notches. The lower intensity dips are from the excitation of higher order WGMs. For the rest of the measurements we concentrate on the larger intensity dips, which correspond to the lowest order (*l* = 1) WGM family, as has been stated by other authors for microcavities based on silica optical fibers^50^.

To extract the Q-factor of the WGMs, the dips are fitted individually with a Lorentzian lineshape; see Fig. 5b,c. As shown later, the measured Q factors of the SH-MOF can vary depending on contact point of the fiber taper and the metal filler, typical Q factors range from 1.1 × 10^3^ to 3.5 × 10^3^. The Q-factors are quite typical for this kind of cylindrical resonators^26,27,37,51^ and the relatively low values are determined by the asymmetry induced on the MOF by the metal filling process. On the other hand, for potential applications the tuning range should be about the free spectral range (FSR)—the spacing of modes separated by a 2π phase difference and given by Δ*λ* = *λ*_*m*+1_ − λ_*m*_. It was found that for the studied microresonators the average FSR associated with the resonant pattern in the specified wavelength range is approximately 3 nm.

**Figure 6:**
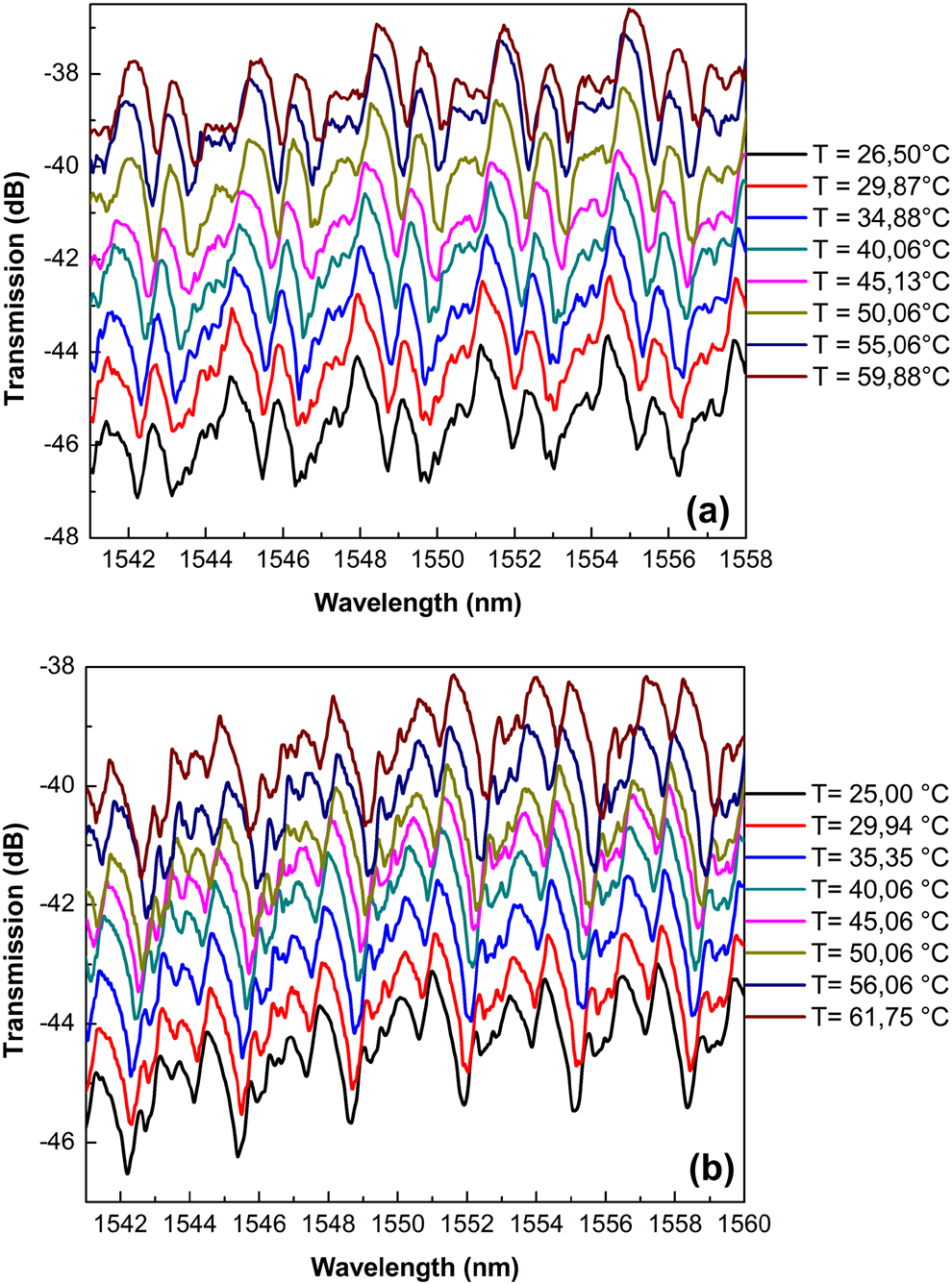
Experimentally acquired WGM transmission spectra at different values of temperature for the cavity based on SH-MOF with Bi electrodes. (**a**,**b**) Thermal tuning process in the TE-polarized WGM spectra with the fiber taper at contact points I and II, respectively. The spectra were shifted vertically for clarity.

Following initial characterization, the temperature response of both cavities was evaluated by monitoring the output spectra at different temperatures. For demonstration purposes, Fig. 6a,b illustrates the changes in the TE-polarized WGM spectra for the Bi-filled SH-MOF microresonator with the fiber taper at contact points I and II under the influence of different values of the temperature. It can be seen that with an increase in the temperature, both spectra experience red shifts. It should be noted that during the experiments, changes in the spectral positions of the WGM resonances corresponding to the variations in the temperature were accurately tracked to eliminate the possibility of ambiguity in determining the wavelength shift direction. As expected, an increase in temperature results in changes in the size and distribution of refractive index within the microcavity, leading to the observed red-shift of the spectral positions of the WGM resonances. It can be clearly seen that efficient splitting of high order whispering gallery resonances does not occur in this case because of the deformation values induced to the MOF^52^.

**Figure 7:**
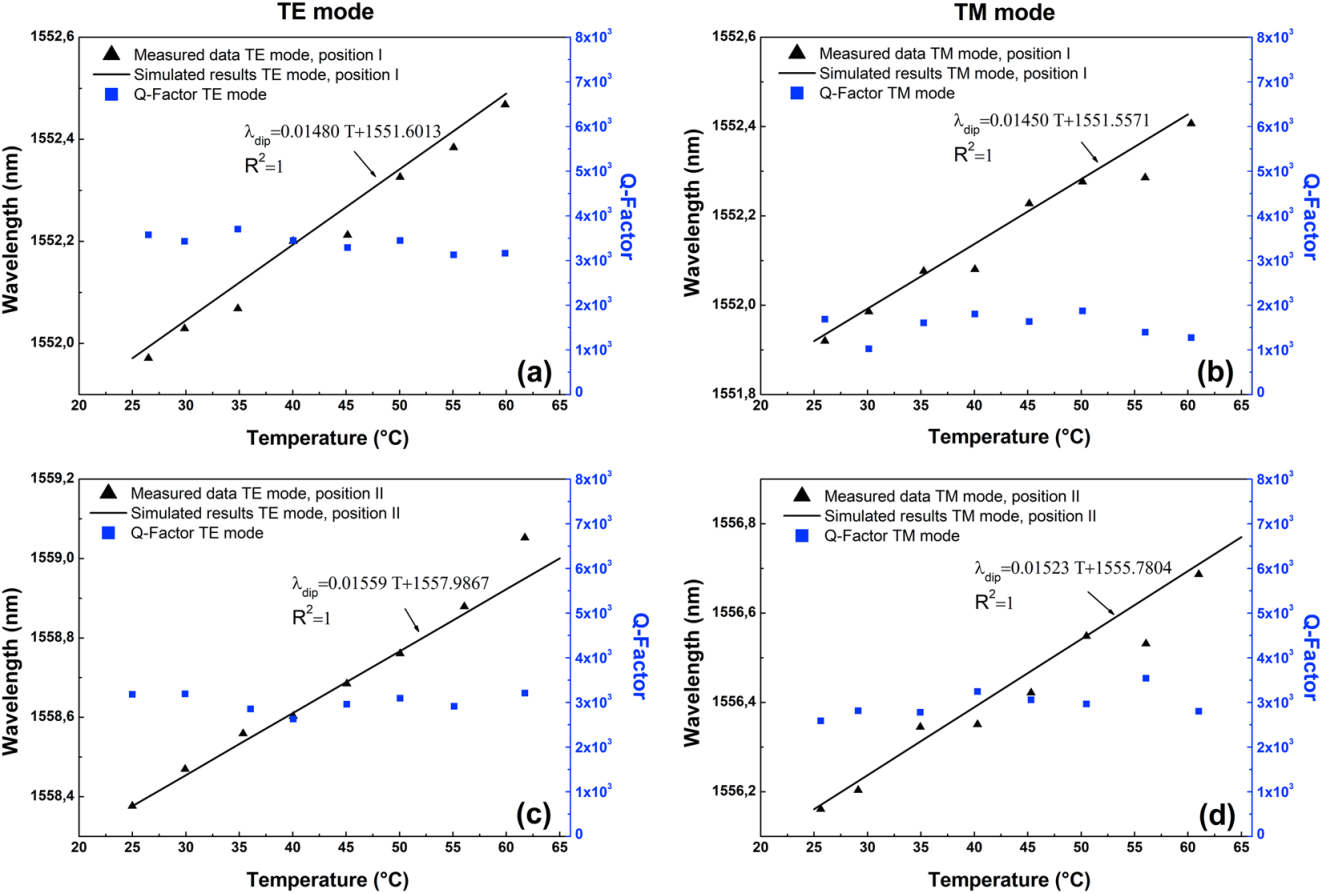
Thermal tuning and Q factor of the microresonator based on SH-MOF with Bi electrodes. (**a**) *TE* mode with the fiber taper at contact point I. (**b**) *TM* mode with the fiber taper at contact point I. (**c**) *TE* mode with the fiber taper at contact point II. (**d**) *TM* mode with the fiber taper at contact point II. The calculation results are in agreement with the experimental results, confirming the observed redshift of the spectral positions of the WGM resonances induced by the expansion of the electrodes with the increase of temperature.

Figure 7 illustrates the dependencies of the four selected *TE* and *TM* WGM resonances for the Bi-filled SH-MOF microresonator versus the temperature value. To study the displacement of the WGM resonances, we select a specific resonance wavelength, for each case, that is close to 1550 nm. For this reason, we follow the resonances at 1551.97 nm and 1551.92 for the ${{\rm{TE}}}_{483}^{1}$ and ${{\rm{TM}}}_{483}^{1}\,\,$modes, respectively, with the fiber taper at the contact point I, while we select the resonances at 1558.38 nm and 1556.16 nm, respectively, for the ${{\rm{TE}}}_{481}^{1}$ and ${{\rm{TM}}}_{482}^{1}$ modes with the fiber taper at the contact point II. Here, the black triangles are the experimental measured data, the solid black lines correspond to fits from the theoretical model discussed above and the blue squares correspond to the experimental Q-factor. There is good accordance between the fits and the different measurement series presented in Fig. 7. As can be seen, for all cases, as the temperature applied increases the WGM resonance wavelength shift increases linearly. However, the sensitivity of the device for the *TE* polarization is higher in each case (14.80 pm/°C and 15.59 pm/°C for contact points I and II, respectively), which, as the analysis indicates, is in agreement with the results of the refractive index changes in Fig. 3 in which for the transverse directions (*n*_*x*_ or *n*_*y*_ depending of the position) the refractive index change in the circumferential region of the cavity is greater in comparison to the longitudinal direction (*n*_*z*_). In that sense, the metal-filled SH-MOF microcavity with fiber taper at the contact point II is more interesting to obtain a photonic device with a greater dynamic range. On the other side, we can see that during the thermal tuning process, the WGM Q-factors do not decrease significantly. Note the slight fluctuations, which mainly result from the unstability of the coupling state between the fiber taper and microcavity.

**Figure 8:**
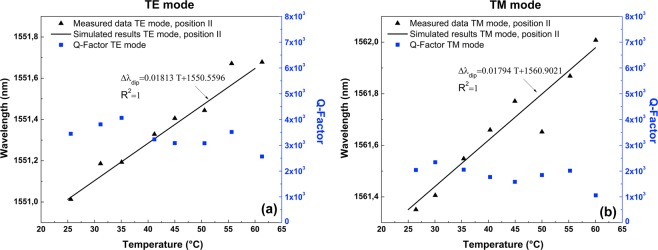
Thermal tuning and Q factor of the microresonator based on SH-MOF with In electrodes and fiber taper at contact point II. (**a**) *TE* mode. (**b**) *TM* mode.

We next outline the effect of the thermal expansion coefficient on the thermal tuning of the proposed WGM photonic device. Here, the experiment only was carried out for the contact point II, considering that the experimental results obtained using the SH-MOF filled with Bi, where it was demonstrated that contact point II is most sensitive to thermal changes and, therefore, where much greater spectral shifts are expected. Figure 8 illustrates the dependencies of the two selected *TE* and *TM* WGM resonances for the In-filled SH-MOF microresonator (at 1551.01 nm and 1561.35 nm for the fiber taper at contact point II for ${{\rm{TE}}}_{484}^{1}$ and ${{\rm{TM}}}_{480}^{1}$ modes, respectively) versus the temperature value. As can be seen in Fig. 8, the thermal tuning also has a linear characteristic and a tuning sensitivity of 18.13 pm/°C for the *TE* mode and 17.94 pm/°C for the *TM* mode in the temperature range from 25 °C to 60 °C. Resonant wavelengths in the In-filled SH-MOF microresonator were calculated using the theoretical model. The calculation results are in agreement with the experimental results, confirming the observed redshift of the spectral positions of the WGM resonances induced by the expansion of the electrodes with the increase of temperature. It is worth noting that with the *TE* mode a maximum WGM wavelength shift of 0.63 nm (0.2 × FSR) at 60 °C is achieved under the current experimental conditions, however the In electrode microresonator is able to achieve 2.63 nm (0.88 × FSR) at the melting point of the metal. In addition, from Fig. 8 it is confirmed, as expected from the theoretical model, that the Q factors are, in general, comparatively lower than those of the Bi electrode microresonator. However, the property of not significantly decreasing the Q factors with the already expected fluctuations due to the unstability of the coupling state between the fiber taper and the microcavity during the thermal tuning is observed.

## Conclusion

In summary, we have proposed and experimentally demonstrated the first tunable WGM photonic device based on microstructured optical fiber with internal electrodes by exploiting the thermal-stress field induced by the expansion of the metal-filled holes. We fabricate side-hole MOF microcavities with Bi and In electrodes to investigate the effect of the thermal expansion coefficient on the thermal tuning of the proposed WGM photonic device. The maximum operating temperature of this tuning scheme is limited by the melting point of the filler metal. Since WGM field distributions are far from the metal-filled holes, it prevents direct interaction between metal electrodes and WGMs, so that the Q factor is not significantly decreased during the tuning process.

Calculations of WGM resonances are performed by using a two-stage computational methodology, where the effects of metal filling process are considered. We demonstrate that the cavity deformation is responsible for the redshift, where the magnitude and distribution of stress are governed by the distribution of the holes in the microstructure and depend strongly on the filler metal. As a result of our study we found that it is possible to estimate the temperature-tuning abilities of the WGM photonic device directly after production, without any previous measurement, just from an image of the SH-MOF cross-section and material parameters.

The metal-filled side-hole MOF platform has remarkable advantages such as ease of manipulation and high tuning sensitivity (18.13 pm/°C for the In-filled side-hole MOF) with a large tuning range (0.88 × FSR), which ensure its potential applications in optical filtering, optical switching, and highly integrated tunable photonic devices.
